# Supplementary material for: Learning massive interpretable gene regulatory networks of the human brain by merging Bayesian networks
Source: PLoS Comput Biol. 2023 Dec 1;19(12):e1011443. doi: 10.1371/journal.pcbi.1011443 (PMC10745139; doi:10.1371/journal.pcbi.1011443)
Supplement: Attachment — Submitted filename: Letter to reviewers-1.pdf [file pcbi.1011443.s001.pdf]

Dear Editor and Reviewers,

Thank you for taking the time to review our manuscript and providing valuable feedback. We appreciate your comments and suggestions, which will help us improve the quality of our paper. We have made the necessary revisions to our manuscript in response to the concerns raised by both reviewers. Please find below our point-by-point responses to each reviewer's comments. We have coloured the original comment in red and respond below in black.

Reviewer #1:

Major Comments:

1) Author's associate KIF17 a gene associated with schizophrenia to KCNIP3 a gene associated with breast cancer. It is not clear if the microarray data used is geared to make this conclusion. KIF17 levels are likely changed by schizophrenia itself, were groups of patients with and without schizophrenia compared to make this conclusion. It is not clear from the study.

We understand the reviewer's concern and apologise for being unclear in the manuscript. Our intention was not to claim a direct connection between the two diseases but rather to demonstrate the potential of FGES-Merge when combined with appropriate visualisation tools to uncover novel gene interactions. We have revised the relevant section of the manuscript (lines 433-439), focusing the figure on how our tool shows the node distributions before and after inference (fig. 6). The comments on disease data have been reduced to just an example on how to add metadata to the nodes for visualisation and a comment in the discussion (also relevant for your minor comment 2) discussing how to use disease state microarray data with FGES-Merge (lines 636-638).

2) Might be worth while to look at unbias function of some the networks found. DAVID and other tools exist where lists of genes can be inputted and top functions are identified.

We appreciate your suggestion to use tools like DAVID to analyse the functional significance of the networks found. However, we realised that the paper was misleading in this respect since the networks shown in the visualisation section are intended as a showcase of the algorithm and the visual tools, rather than to extract biologically relevant results directly. We have restructured the paper to put more focus on our contributions so they are clearer from the beginning and we hope the change of figure 6 helps avoid this confusion in the future.

Minor Comments:

1) Figures are hard to navigate. There is a lot of color used and it is difficult to interpret what they mean if any.

We apologise for the confusion caused by the initial presentation of our figures. We have revised the figures, specially those in the visualisation section (pages 12-15) which we believe were the most confusing. We have removed some of the least useful layouts from figure 4, changed figure 6 as discussed above and tried to make the captions of figures 5 and 10 clearer regarding the use of colour.

Reviewer #2:

We wanted to thank Reviewer #2 for believing our work to be important enough to merit such a thorough review which led us to do a major rewrite of multiple sections of the work. We think his comments have helped us improve the presentation and discussion of our results. We have tried to address all the points raised to the best of our ability and we hope we have succeeded in meeting his expectations.

Main comments

it is not clear whether a 'genome-scale' GRN is truly informative. A GRN will be useful in deciphering molecular mechanisms during a given biological process (your subnets in the brain gene expression analysis). An overarching GRN is likely an utopia. Mechanisms are not activated all at once. Even if there was a one encompasses them all GRN, only subsets, possibly with different relationships (and some common OK) would be visible in clearly defined conditions. This needs to be clearly explained. For example, the use of 'averaged' gene expression to reconstruct the global GRN doesn't seem to make sense. Think of the local interaction between two genes that could be very different between two conditions (e.g. cancer or healthy patient). What would the 'average' represent?? Same for different brain regions.

We agree with the reviewer that given the assumptions we make and the limitations of the data, we can't guarantee that the relationships we find between genes can be interpreted directly as regulatory interactions and so the genome scale network might not be informative in the common way GRNs are used. We have also stopped using the word "averaged" since we think it is misleading and a mistake on our part. We were trying to refer to the fact that each sample comes from a population of different cells in the tissue, so the expression is not representative of any single cell in the population. However, we still believe that FGES-Merge lifts a meaningful technical limitation and provides a useful tool for comparing the learned structure of genome-sized networks in different contexts (e.g. diseased vs healthy, or different tissues). To address this issue we have:

1. Emphasised that the objective of learning the structure for a genome wide network is to showcase the ability of FGES-Merge to scale to tens of thousands of variables (changes in the abstract, lines 19-23 in the introduction, lines 541-544 in the Human Brain Regulatory Network section)
2. Added a paragraph in the discussion addressing directly the limitation of using microarray data (lines 627-638)
3. Emphasised a possible application of genome-sized networks by using our visualisation tool to compare the structures learnt across different contexts (different brain structures) to highlight the differences (lines 570-574, 637-638).

Avoid referring to the challenge data you use as the DREAM5 challenge data set. The latter was concerned with Genetical Genomics data (marker and expression data), see https://www.synapse.org/#!Synapse:syn2820440/wiki/71025. I agree that the Network Inference challenge you work on is coined DREAM5 by the Marbach et al. paper though.

We have also clarified the name of the challenge data we use. There were multiple challenges for DREAM5 (four in total, as listed on their website https://dreamchallenges.org/?s=dream+5), and the challenge we used is the Network Inference Challenge, not the Systems Genetics Challenge. To avoid confusion, we now use the full name of the challenge the first time we refer to it (line 29), and we

provide a link to the challenge description and data in line 490. Subsequently, we refer to the challenge as the Network Inference Challenge.

Some statements need at least a reference, see minor points below. More generally, introduce what you need and justify it bullet-proof, otherwise it's speculations. At best can be discussed. And your discussion section is very scarce. Actually more a quick conclusion. Might repeat myself, but very little additional work, a clearer organisation of the argument, would convince your readership so much better, make that communication.presentation effort, your work is worth it!

Following this comment and the requests for clarification from some of the minor comments, we have majorly restructured the paper to hopefully make everything clearer to the reader. We will comment on the biggest changes:

- We have removed the section on advantages and limitations of BNs and the problem statement. We believe they were not very clear and that everything in them was better explained elsewhere in the manuscript. Our interest in using BNs for explainability is now presented in the introduction (lines 14-23) and the limitations of the method are raised as we present the theoretical background and discussed in their own section at the end of the manuscript (pages 22, 24). .
- The theoretical background has been restructured completely. We now start by presenting Gaussian Bayesian networks: We introduce the problem of learning a BN from data and what we can do with a BN once we have it (lines 46-76). We explain GBNs and the advantage they have for parameter estimation and inference in massive networks (lines 77-116). Then we go deeper into the problem of learning the structure of a BN from data, introducing score-based methods and giving some context for our contributions. (lines 117-142)
- We have reduced the discussion on gene regulation, trying to focus only on what we need for FGES-Merge. We briefly explain gene regulation and only microarray data collection, we then introduce GRNs as a way of modelling gene regulation and directly relate them to BNs (lines 143-183).
- We have rewritten the section on the Human Brain Regulatory Network explaining the reasons for using the human brain as a showcase and why we make a network for the whole genome. The context in which FGES-Merge has been developed was missing from the previous version of the paper and we believe it helps explain some of our choices (lines 538-553).
- We have added a discussion section commenting on all assumptions and limitations of FGES-Merge, considering which of them are necessary for the algorithm to function and which can be relaxed. We also introduce some avenues of future work that could improve the limitations of FGES-Merge (lines 593-638).
- We have removed the Appendix with the overview of other methods for learning GRNs and only covered what we needed in the main text.

Minor comments:

We have addressed all comments about typos and general editing. Below we respond to all other comments:

l5 'single-cell' -> will you use single-cell data? If not, remove, a general remark, simplify your work, do not get encyclopaedic, stick to what is of interest to *your* work.

l5 "most of the information about the development and function..." -> dangerous sentence. What about epigenetics? Genome plasticity?

We have removed these sentences and in general rewritten the introduction and the section on gene regulation to emphasise that our contribution is algorithmic and avoid making any sweeping statements about a topic in which we aren't experts. We briefly mention post-transcriptional modifications in line 10 and non-genetic factors of regulation in lines 150-152. We mention single cell data only to mention that microarrays sample populations of cells instead (lines 165-168). We have added some thoughts to the discussion on adapting the model to learn networks that take into account those factors if data is available (lines 612-616)

l55 "the model what model?? Also check the reference's relevance on l56

l66: ref [14] doesn't have interactions

Removed any reference to a model. In the section of the revised work we present the assumptions of non-genetic factors only contributing indirectly and steady-state directly as simplifications of the problem of understanding gene regulation. (lines 149-155). We have revised and modified the references in line 155.

l77 so what does the measurement represent? How to compare samples?

To the best of our knowledge, significant differences in the levels of expression between samples represent differences in the underlying cell population or in gene regulation at the time the sample was taken. As the reviewer mentioned, not all pathways are active at the same time, so this might be the cause of the differences. By studying those differences more closely we can try to understand if they are due to functional differences between the tissues from where the samples were taken.

l96 as you pointed out, a precise definition is key. Still waiting for yours at this stage

We go over the interpretation of the edges in a GRN modelled by a GBN in lines 189-190. Each edge represents a probabilistic relationship between the nodes. More concretely, A -> B implies that the probability distribution of B depends on the values of A. This concrete dependence is explained now more clearly in lines 89-91 and Eq. 3.

We are not sure if the reviewer was referring to this.

l129 no, this does not imply that the connected components (cc) are dense!

That was a mistake, thank you. We have removed this line. The implication was backwards and, even then, not correct. The existence of hubs and the fact that the network is globally sparse implies that to compensate for the large number of edges some nodes have, some other nodes will have less edges than the average of the network, which gives a large number of disconnected or very sparsely connected nodes. This means that the networks we learn will be very small compared with the maximum size most of the time.

l133 a ref (local sparsity requirement)?

The way sparsity is introduced in the FGES algorithm is by assuming the number of parents of each node will be small, this is the local sparsity requirement. It's very common in the literature of

Bayesian networks to use “sparse network” to refer to “the maximum in-degree of every node is a small constant number, much smaller than the size of the network” which is our local sparsity requirement when in general computer science and elsewhere “sparse network” means “the number of edges is much smaller than the maximum possible”. We introduced the global and local sparsity distinction to avoid confusion and to clarify why, even though the network is “sparse” (in the second sense), we can’t use the usual methods that apply to “sparse” (in the first sense) networks. Since the distinction is not standard in the literature we don’t have a reference to add.

l142 definition of 'self-similar'?

We have rewritten the sections on in-degree and out-degree from the topological properties of GRNs (lines 215-242) and now refer to the distributions as following a power-law instead of using “self-similar” which could be confusing. We give a concrete formula that approximates the in-degree distribution. (Eq. 10) We hope that the rewriting clarifies that we use the topological information here to restrict the size of the subnetworks and to explain how we use direct edges finding hubs.

l195 [27] is NOT "very" recent. Sometimes a general issue in your work, you rely on some old references in a very quickly moving field.

We have fixed that line. We have also tried to update our review of methods for structure learning with ref. 12 and have added some more recent work to our discussion with refs. 45 and 46 discussing the problems with assuming Gaussianity (45 discusses the importance of knowing the actual shape of the distribution at each gene and 46 is recent work for scalable BN learning and inference for more general distributions. We are currently working on adapting FGES-Merge with the work done by 46.)

l204 conditional independence -> some algorithms ignore this (score-based)

This section has been removed and we avoid discussing constraint based algorithms in the section on structure learning since we don’t use them (starting line 117). However, even when using score-based methods, the definition of a BN means that the structure encodes information about conditional independence between the nodes.

l208-209 try and be more precise. Maybe even accurate.

This section has been removed. We explain inference in general in lines 71-75 and 103-116 and have clarified the example given in the visualisation section (fig. 6).

l211 "output at each node" -> ??

We were referring to the CPD associated with each gene. We have removed this section and made it clearer how we model a GRN using a BN, including the meaning of each edge and node in lines 182-183.

l219 the [brain] is "not so well studied" -> so why using it for your showcase?? Not logical.

We were referring to the fact that not having expert knowledge to restrict the space of possible structures meant that algorithms that could explore a high-dimensional space would be required. This

was very unclear from the text, so we have cut it. Our motivation for using the brain is covered in the Human Brain Regulatory Network section (lines 539-544).

l253 computational cost an issue OK, more more fundamentally: the high-dimensional aspect of the problem. Needs to organise your thoughts on those issues, they appear here and there in the manuscript.

This section has also been removed. Our general interest in solving high-dimensional problems while maintaining interpretability is now mentioned more explicitly in the introduction (lines 14-19). The fact that the problem of learning the structure of a BN is NP-hard is introduced in lines 69-71 alluding to the high-dimensionality of the search space and mentioned again when discussing the choice of algorithm in lines 126-129.

(l256) Clarify again the different nets for different regions (aka conditions) vs a unique global network.

This is now discussed in the Human Brain Regulatory Network section. The networks for different regions are obtained by only using samples from those regions filtered according to the anatomical structures defined by the EBRAINS atlas (line 549-550). These are used for comparison to obtain differences in expression between regions (lines 570-574). A global network is learnt from all data and on its own is not as interesting, but can be used to compare with other global networks learnt from other individuals (that could be ill and so the networks could be compared to obtain differences in a similar way).

(l259) Examples of 'other questions that might..."

We now go over some of them in the discussion of future work (lines 614-619). Mainly, adaptations of FGES-Merge to deal with dynamic data or the introduction of non-genetic regulatory factors, which would increase the dimension of the problem even when dealing with less than genome-sized networks.

l270 give the general concept of your novelty

We have removed the section on the problem statement. BayeSuites' main innovation is in presenting a complete web tool for learning and visualising Bayesian networks which was not available before. We mention this in the introduction of the section and have added a reference to a stand-alone paper with more detail about BayeSuites (lines 391-396).

l299 local BIC -> why this choice? Motivated later in the text only. Have you compared your approach to that using the MI? Also in Fig 1, refer to text when mentioning BIC (self-sufficient captions).

l310-315: order in the logical flow?

BIC and MI are both measures of the strength of the relationship between two variables. We used BIC because it was computationally expensive to calculate both and because our model assumes linear relationships between the variables, so the main advantage of using MI is lost. This is now explained in lines 302-309. We have also fixed the caption of Fig.1, adding eq. 11 directly and not referring to the text.

l305 -> well I would disagree, what about single-cell data you mention? More like small counts. Also in Appendix A "although an estimate can be found but with no guarantee of correctness" -> wrong, lots of research work has gone in this direction! Revise your arguments and make justice to people working on theoretical aspect of high-dimensional problems.

l309: what kind of inference? Probability queries? Where does this plug-in, it seems to come on top of an already very complex setting.

This has all been removed as part of the restructuring of the theoretical background. The assumption of Gaussianity, the definitions in the appendix and inference are now presented earlier to avoid complicating the explanation of the algorithm. The appendix has been removed with only the sections required to understand the algorithm introduced in the theoretical background.

l351: this would be an ideal behaviour. What guarantee do we have this will occur?

The probability of choosing a random edge depends on how big the difference in score is between the best one and the randomly chosen one and a parameter of the algorithm (the temperature in simulated annealing), that we make proportional to the size of the network. This parameter decreases as edges are added so that by the time the network has as many edges as nodes we never choose a random edge. This is a typical simulated annealing setup. We have added a reference to the line on simulated annealing (line 350).

l356 "original" refers to? l357, isn't the argument upside-down?

With "original" we are referring to the network containing all genes. We have rewritten this line. The argument is that by allowing for suboptimal edges we increase the number of edges in the final network and make it more likely that the actual optimal edges will be available. Since after merging we can only eliminate, not add, edges we want to err on the side of adding too many. This is better explained now in lines 363-367.

l374 improved over [26]? Careful in re-using challenge data sets and performing better? It is considered as a sort of over-fitting since you know the results, hence optimise your method accordingly in a sense. E.g. one could argue you use the MCC criterion instead of the AUPR to show improved performance.

l536 How about varying the s_{max}'s? (for calculating the AUPR)

We have explained in the revised text that estimating the AUPR in this way would have been computationally infeasible and that's why we use the MCC (lines 502-509), but we acknowledge that by changing the score and reusing challenge data we are more likely to overfit our method to the challenge. We have updated the text to reflect that our interest is in comparing our performance to other methods to see that we are at least comparable to the state of the art while reducing the time taken to learn the structure, not claiming to be better at this concrete task (lines 520-522, Fig.8).

l391 Ref for equations in "Inference"?

We have clarified that we follow Koller D, Friedman N. "Probabilistic Graphical Models: Principles and Techniques" for the equations about BNs (line 49-51).

Eqn (6) etc. What if E is not included in Pa(Y)? Seems clearer later, but was wondering what you were doing at this stage.

E can be a set of variables not in the parents of Y. If they are not, the betas encode the influence of the evidence, E, on Y through its parents. We have added a few lines explaining the procedure before giving the equations in lines 109-112 and have changed the notation to make it clearer that the betas here (Eq5-8) are not the same as the betas in equation 3.

l444-45 a reference? Why show unuseful layouts in Fig. 4? l454 a reference?

We wanted to show all available layouts in the tool, but we have reduced the number of figures to only show the force directed and the tree layouts. The recommendation of force directed layouts was ours from trying them on our networks. We have made this clearer and explained our reasoning (lines 442-445).

l458 does the data reflect this?

We aren't sure about what the reviewer is referring to here. We are adding metadata to the nodes so that each node has a list of conditions it's related to and the visualisation can use those conditions to change how it shows the nodes. In this case, we have added information from the DisGeNET database about the conditions to which each gene is related and we use the first condition in each gene to colour them. (Fig. 5)

l468 parents observed at average value seems very crude, so no visualisation of interactions?

Interactions can be seen from the edges of the network. The effect of each parent on the child can be seen from inference, as in fig. 6. We have clarified in the text that the marginal distribution is the expected distribution of the level of expression of the node if we don't know anything else about the expression of any other gene (we had previously made a mistake, the average of the marginal distribution is the same as the average of the conditional distribution if the parents are observed at their average value due to the properties of linear Gaussian distributions, however the standard deviation is different) (lines 444-446).

l478 'original joint distribution' yet another example of a conceptused before it is clarified.

We now introduce the joint distribution and how to construct it from the BN in lines 88-92. We explain that inference can be done in closed form using the joint distribution in line 94 and go into more detail in lines 103-116. We have rewritten this part to make it clearer what the joint distributions we are referring to are (lines 455-456).

How do you compare distributions? The Kullback-Leibler divergence is a classical ones. Others have been used…

For visualisation of GBNs we don't think it's necessary to use KL divergence or other similar metrics for comparison. Since the shown distributions are always univariate Gaussians all the information needed to compare them is in the means and standard deviations, which are shown. We also think that visual inspection of the differences is much clearer than giving a score.

This would be more interesting for multivariate distributions (and can easily be done programmatically from the network learnt by FGES-Merge) but for our visualisation tool we are limited to univariate ones due to how hard it is to visualise distributions in more than two dimensions.

l523 GG entries -> explain the notation

This is a Latex error. It was meant to say G x G entries, as in one entry for each possible edge in the network (line 494).

Fig. 7 (d) seems to indicate little overlap. Comments?

Yes. In general the structures recovered by the Network inference Challenge methods were poor and ours is not an exception. This is what we expect to see given the scores in Fig 8a.

l538 argument: well biologists could test according to the ranking of edge likelihood. And/or their prior interest in some genes/relationships/

Agreed. This was a poor argument and was ignoring the fact that in the end there is an implicit probability and threshold for each edge in our method too, even if it can't be easily extracted. In the end a matrix of probabilities for each edge is the best we can do. We have removed this from the text.

l555 say more explictly 1 and 2 refers to the BN methods labelled Bayesian1/2 in your plots

We have added a table (Table 1) explaining all other Bayesian network methods in the challenge and have removed references to individual methods and "doing better" or "worse" as discussed above with the paragraph about MCC and AUPRC.

l562-564: very thin argument.

Agreed. We have removed it. Even though there might be some differences in the topological properties of GRNs in prokaryotes and eukaryotes and that might lead to better or worse performance for our algorithm, we have not tested this claim. And the differences in topological properties between species might be much higher than between eukaryotes and prokaryotes for all we know.

l568 GENIE3 is more a tree ensemble method. Yes relying on a regression setting, but so is yours in the end.

We have corrected this and explained better why we chose GENIE3 (its complexity is at worst quadratic in the number of genes and it did well in the Network Inference Challenge) in lines 527-530.

l589 not clear what is done to obtain this series of GRNs. Why not pool those, it seems to be what you announced. Then you could analyse those. No real validation, but from the global topology perspective. Any publication on link? Overlap with know data bases, e.g. STRING?

l595 See e.g. https://academic.oup.com/bioinformatics/article/25/3/417/244497, some work make a better use of different yet related conditions. Average gene expression (l603) seems VERY dubious.

We've removed the "averaged" gene expression since it was not clear from the text and we have tried to clarify how we learn the networks (lines 547-550). (We filter the samples by region of the brain and

use FGES-Merge on the samples to build a network for each region and one with all the data available.)

l607-608 how is this pruning performed?

This is the normal pruning step of FGES-Merge. We have clarified that what we mean is that by the topological properties of the network we expect that the end result will be globally sparse so we place a limit on how many edges we can have. If there are more than that we remove the worst performing ones. We realised that this was not explicitly in the text in the original manuscript, only vaguely hinted at in the caption of fig. 3. We have added it to the text in lines 363-367.

l627 give counts of other nodes. 80 parents is HUGE Is this realistic?? The average value is quite high too.

l637 the nodes WITH PARENTS

l641 too dense? Also l642-643 are not convincing (me).

We have changed the figures in this section to two clearer histograms for the out-degree and i-degree of the network with the power-law distributions that best fit the data so that it's clearer what fraction of nodes have a given in- or out-degree (Fig.10). We agree that the numbers are relatively big. There is a trade-off when exploring using simulated annealing and pruning and we think our current hyperparameters err to the side of a higher false positive rate (lines 583-592). Again, we have de-emphasised the accuracy of reconstruction for the Human Brain Regulatory Network, since we now believe that the question was ill-posed. We briefly discuss the topology and remark that the main objective of the network is to showcase the ability of FGES-Merge to scale to that number of variables and of BayeSuites to visualise and manipulate the network while a better benchmark for our ability to recover the underlying structure of gene regulation are the networks from the Network Inference Challenge.

Would be good to have a discussion. Clearly missing. Sometimes, embryos are present in the results.

l648 yes the absolute scores are low. But what do they mean? If this gives enough new knowledge for future research, this is already a win. On the other hand, what if we hit a glass ceiling? Discuss!

We hope the new discussion section helps the reader see what our thoughts are on how FGES-Merge can be improved and what uses it could have for research. We have moved way from discussing accuracies or scores in the reconstruction of the network since we don't think our tests are comprehensive enough and place the focus more on extending the algorithm to account for more types of data (heterogeneous sources and dynamic data) which we believe might be available in the EBRAINS Atlas by the time FGES-Merge is deployed.

l663 queries such as? Not demonstrated in your work

The possible queries are hopefully better explained now with the rewritten section on inference and the changes in the visualisation section and Fig. 5.

We hope that our revisions have adequately addressed the concerns raised by both reviewers. We believe that the updated manuscript is now significantly improved and more suitable for publication. Thank you again for your valuable input, and we look forward to your feedback on the revised manuscript.

Sincerely, on behalf of all the authors,
Niko Bernaola
